# A Model of an Integrated Immune System Pathway in *Homo sapiens* and Its Interaction with Superantigen Producing Expression Regulatory Pathway in *Staphylococcus aureus*: Comparing Behavior of Pathogen Perturbed and Unperturbed Pathway

**DOI:** 10.1371/journal.pone.0080918

**Published:** 2013-12-06

**Authors:** Namrata Tomar, Rajat K. De

**Affiliations:** Machine Intelligence Unit, Indian Statistical Institute, Kolkata, India; University of Iowa, United States of America

## Abstract

Response of an immune system to a pathogen attack depends on the balance between the host immune defense and the virulence of the pathogen. Investigation of molecular interactions between the proteins of a host and a pathogen helps in identifying the pathogenic proteins. It is necessary to understand the dynamics of a normally behaved host system to evaluate the capacity of its immune system upon pathogen attack. In this study, we have compared the behavior of an unperturbed and pathogen perturbed host system. Moreover, we have developed a formalism under Flux Balance Analysis (FBA) for the optimization of conflicting objective functions. We have constructed an integrated pathway system, which includes Staphylococcal Superantigen (SAg) expression regulatory pathway and TCR signaling pathway of *Homo sapiens*. We have implemented the method on this pathway system and observed the behavior of host signaling molecules upon pathogen attack. The entire study has been divided into six different cases, based on the perturbed/unperturbed conditions. In other words, we have investigated unperturbed and pathogen perturbed human TCR signaling pathway, with different combinations of optimization of concentrations of regulatory and signaling molecules. One of these cases has aimed at finding out whether minimization of the toxin production in a pathogen leads to the change in the concentration levels of the proteins coded by TCR signaling pathway genes in the infected host. Based on the computed results, we have hypothesized that the balance between TCR signaling inhibitory and stimulatory molecules can keep TCR signaling system into resting/stimulating state, depending upon the perturbation. The proposed integrated host-pathogen interaction pathway model has accurately reflected the experimental evidences, which we have used for validation purpose. The significance of this kind of investigation lies in revealing the susceptible interaction points that can take back the Staphylococcal Enterotoxin (SE)-challenged system within the range of normal behavior.

## Introduction

Thymus derived lymphocytes/T cells play a central role in cellular immunity [1]. Naive T cells (that have never encountered antigen) circulates between peripheral lymphoid tissues (*e.g.*, lymph nodes) and can detect the specific antigens present on the surfaces of dendritic cells. Encounter with a specific antigen leads to the activation, proliferation and differentiation of T cells into mature/memory T cells [2]. Stimulation through T cell receptor (TCR) can lead to distinct responses in naive and memory CD4 T cells [3]. Peptide antigen stimulates both naive and memory T cells, whereas, soluble anti-CD3 antibodies and bacterial/viral superantigens (SAgs) stimulate only naive T cells to proliferate and secrete cytokines. Naive T cells lead to a vigorous proliferative response to superantigen (SAg) stimulation *in vivo*, which is followed by a partial deletion of responder T cells. Responder T cells that survived the SAg response were found to be incapable of generating a secondary proliferative response to a SAg challenge [4].

**Figure 1:**
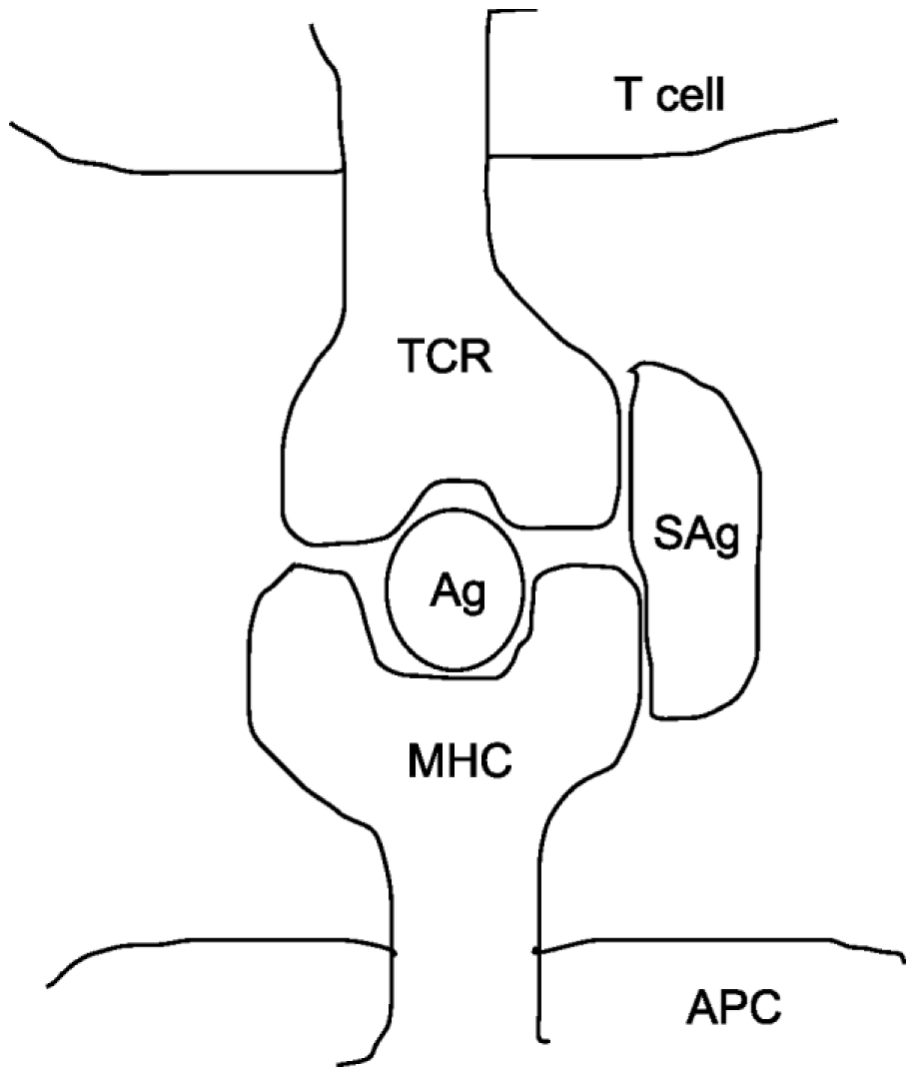
A schematic model of SAg of *S. aureus* interaction with TCR and MHC class II molecules of an infected *H. sapiens*. Here, one can find the difference between antigen and SAg binding site on TCR.

*Difference between conventional antigen and superantigen*: SAgs are bacterial and viral proteins that are potent activators of T cells. Conventional antigens are first processed within antigen presenting cells (APCs), bind with the peptide binding groove of MHC, and are presented to T cells with an antigen specific T cell receptor (TCR). In contrast, SAgs skip these processes. SAg proteins are usually of bacterial or viral origin, which are not internalized by APCs. They do not undergo intracellular processing, and are not presented on the surface of APCs [5]. Escaping these processes, SAgs bind directly to MHC, outside the peptide binding groove. It is shown in Fig. 1. Due to this, they bypass MHC restriction and TCR specificity. This SAg binding mechanism activates a large proportion of T cells (up to 25% of all T cells) in comparison to conventional antigens (0.0001% of all T cells) [6], [7]. SAg interacts with the variable region of TCR-V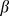 chain [8]. Therefore, SAg recognition by T cells has a significantly lower degree of specificity, as compared with their interaction with conventional antigens. This is the another reason that explain the property of SAgs to stimulate a large number of T cells. *Staphylococcus aureus* can cause severe toxic shock through the release of pyrogenic exotoxins, known as SAgs, which trigger massive T cell activation and a ‘cytokine storm’ [9]. A brief description about the model organism and the regulation of its SAg expression regulatory pathway has been also discussed.

*Superantigenicity of Staphylococcal Enterotoxins (SEs)- reason behind the choice of SAg for designing the study*: SEs exert their effect on the host organism as follows: (1) as enterotoxins, they induce emesis and diarrhoea in humans and non-human primates [10]; (2) as exotoxins, they induce toxic shock [11]; and (3) as SAgs, they induce V/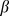-specific T cell stimulation [12], followed by anergy and activation-induced cell death (AICD) by apoptosis. The clinical manifestations of SEs intoxication are associated with the massive proliferation of T lymphocytes that lead to the large-scale release of pro-inflammatory cytokines, *e.g.*, interleukin 2 (IL-2) and tumor necrosis factor 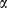 (TNF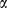) [13], [14]. SEB reactive CD4 T cells and CD8 T cells mounted a strong proliferative immune response after primary SAg challenge. However, they failed to expand in secondary challenge [15], [16]. They (specially Staphylococcal enterotoxin B (SEB)) normally exert their effects on the intestines and thus are termed as an enterotoxin. SEB has been studied as a potential biological agent of war, since it easily can be aerosolized, very stable, can cause widespread systemic damage and multiorgan system failure.

*Pathogen perturbation on host system*: In order to evaluate how SAg affects the TCR signaling pathway, it is first necessary to understand the dynamics of a normally functioning pathway. It can be used as a baseline against which a pathogen perturbed system can be compared. Such comparisons can expose the most susceptible proteins that are altered by a pathogen as well as the most critical reactions responsible for better functioning of a host’s biochemical network. In general, the outcome of host-pathogen interactions (in terms of a naive infected host) is dependent on the balance between the host immune defense and the virulence of a pathogen.

There exists a few *in silico* studies on host-pathogen interactions. FBA has been coupled with experimental studies to predict how viral infection would alter bacterial metabolism [17]. Two separate networks of interactions have been synthesized between host immune components and two closely related bacteria of the genus *Bordetellae*, *viz.*, *B. bronchiseptica* and *B. pertussis* [18]. The model indicates that the infection time course of both *Bordetellae* can be separated into three distinct phases based on the most active immune processes. Co-culture experiments were performed for clinical strains of the opportunistic human pathogen *Pseudomonas aeruginosa* with *Dictyostelium* amoebae, to investigate the interactions of two organisms, such as, bacteria’s pathogenic action against amoeba [19]. A mathematical model was also developed to infer how the populations of the amoeba-bacteria system evolve considering the features of this interaction.

FBA has been used to analyze genome-scale reconstructions of several organisms; it has also been used to analyze the effect of perturbations, such as *in silico* gene deletions. The current state of the art for linear optimization in FBA is limited to the optimization of single objective function. We have considered modified methodology of FBA [20], [21]. For the current study, we have also considered two conflicting objectives, *viz.*, minimization of toxin expression in a pathogen (*S. aureus*) and maximization of the concentrations of a stimulatory molecule present in TCR signaling pathway of an infected host (*H. sapiens*) for a case. Our aim is to compare the pathogen perturbed host system with that of unperturbed one.

First of all, we have designed TCR signaling pathway of *H. sapiens* as given in literature and different biochemical pathway databases, then integrated it with SAg expression regulatory pathway of *S. aureus*. We have studied and compared the behavior of TCR signaling pathway in six different cases: (1) We have first implemented the methodology over an unperturbed TCR signaling pathway of *H. sapiens* and observed the concentrations of regulators (*e.g.*, addition of phosphate group for phosphorylation reaction) that maintain the required status/concentrations of the particular signaling molecules, (2) Secondly, we have studied the pathogen perturbed TCR signaling pathway (an integrated pathway of SAg expression regulatory pathway of *S. aureus* and TCR signaling pathway of *H. sapiens*), where objective function was to maximize the concentrations of stimulatory molecules (ZAP70, LCK and FYN) form. (3) In the third case, we have maximized both SAg expression in *S. aureus* as well as the concentrations of stimulatory molecules (ZAP70, LCK and FYN) to study the behavior of perturbed TCR signaling pathway molecules. (4) We have studied the behavior of the same integrated pathway on the optimization of two conflicting objective functions under FBA, where minimization of SAg expression and maximization of the concentrations of stimulatory molecules (TCR:CD3 complex and ZAP70) have been done. (5) In case (5), we have reversed the conflicting objective functions for optimization as given in case (4). (6) In the last case, the change in the concentrations of some of the signaling molecules, *viz.*, ZAP70, LCK, FYN is explored, under pathogen perturbed and unperturbed conditions. We have compared the computed *c*-values and found that they were lower for most of the signaling molecules present in an unperturbed TCR signaling pathway in comparison to the perturbed one, except for the signaling molecules, *e.g.*, SHP1 and CBL, which keep the TCR signaling pathway in a resting state. Moreover, for the last case (6), we have observed the higher concentration levels for the regulators that maintain status/concentrations of CBL and SHP1 and lower for immune response molecules, upon minimization of the concentrations of molecules, ZAP70, LCK and FYN in an unperturbed TCR signaling pathway. The results were in contrast to this for the case, where maximization of the concentrations of ZAP70, LCK and FYN in a perturbed TCR signaling pathway, has been done. We have compared the simulation results with existing experimental evidences and depicted the similarities between the two for validation purpose. We have also generated a hypothesis based on the *in silico* results. This kind of study can reveal potential targets for drugs also.

### A Brief Description of the Pathways Under Integration: An Integrated SAg Expression Regulatory Pathway in *S. aureus* and TCR Signaling Pathway in *H. sapiens*

Here we describe the pathways which are integrated for the study, *i.e.*, SAg expression regulatory pathway in *S. aureus*, TCR signaling pathway in *H. sapiens* and molecular interactions among them. We have been able to construct an integrated pathway model for host-pathogen interaction based on the information available in literature as described below.

### SAg Expression Regulatory Pathway in *S. aureus*

**Figure 2:**
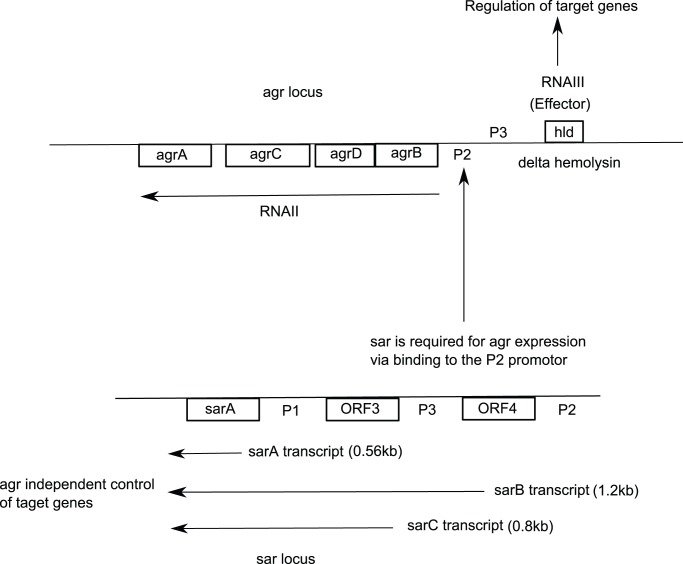
agr and sar loci from *S. aureus*, adapted from [140].

*Staphylococci* are Gram positive cocci of 0.5∼1.5 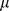
m that constitute a considerable part of the normal skin-flora of humans and several different mammals. At least six global regulator loci (agr, sar, sarH1, sae, rot, 1E3) have been identified. Two of the regulatory loci have been cloned, sequenced in detail: agr (accessory gene regulator) and sar (staphylococcal accessory regulator). These loci has been shown in Fig. 2. The global regulatory locus agr consists of genes agrD, agrB, agrC, agrA, hld. It encodes a quorum sensing system involved in the generation of transcripts, RNA II and RNA III from two distinct promoters P2 and P3. The P2 promoter derives RNA II molecule, encoding an operon containing, agrBDCA, with AgrC as the sensor protein and AgrA as the activator protein of the two components regulatory system. The membrane located AgrC is activated by an octapeptide pheromone encoded within the agrD gene, known as AIP, for autoinducing peptide [22]. AgrD is modified and secreted through the involvement of AgrB protein [23]–[25]. The system is double autocatalytic in the sense that it produces its own transcriptional activator (AgrA) and its own inducing pheromone (AIP). Induction of agr system leads to activation of the RNAIII gene that is the actual effector molecule of the agr dependent virulence gene regulation [26], [27]. It also encodes for delta hemolysin (hld).

**Figure 3:**
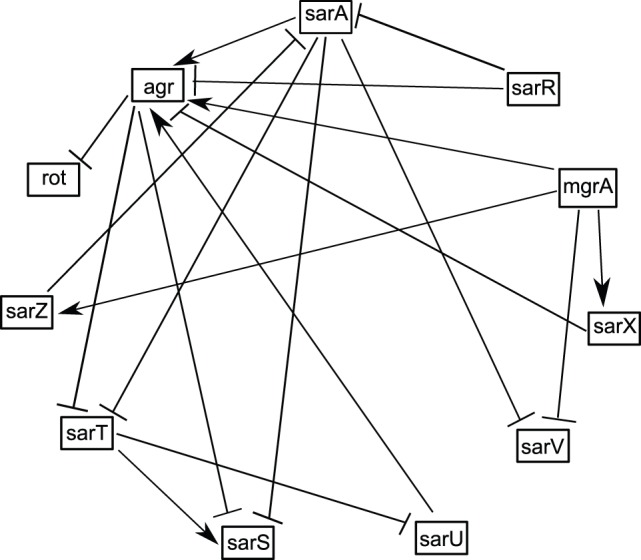
Gene regulatory network involving sarA family, agr and rot (transcriptional regulators) in SAg expression regulatory pathway of the *S. aureus* [27], [31]. Sharp arrow head indicates activation and blocked arrow head shows inhibition.

A second regulatory locus with pleiotropic effects on the production of virulence genes is sarA (staphylococcal accessory regulator) [28]. Unlike agr, the sar locus activates the synthesis of both extracellular (*e.g.* hemolysins) and cell wall proteins (*e.g.*, fibronectin-binding protein) in *S. aureus*. The sar locus, contained within a 1.2-kb1 fragment, is composed of three overlapping transcripts designated sarP1 (sarA, 0.56 kb), sarP3 (sarC, 0.8 kb) and sarP2 (sarB, 1.2 kb). SarA protein level is an important determinant of agr activation [29]. agr and sar loci effect the regulation of many virulence genes, like, seb, sec, hla, hld and TSS1 among others [30], [31]. It has been demonstrated that in agr mutant strains, mRNA steady-state levels were reduced 4-fold for seb, 5.5-fold for sed, and 2 to 3-fold for sec [32], [33].

sar was subsequently changed to sarA based on identification of 11 homologs [34]. Members of the SarA family interact with each other to form a complex regulatory network controlling virulence factors. These can be divided into three structural families consisting of (1) single-domain proteins- SarA, SarR, SarT, SarV, SarX and Rot; (2) larger, two-domain proteins- SarS, SarU, and SarY; and (3) small homologs with similarity to the MarR protein of Gram negative bacteria, *viz.*, SarZ and MgrA [34]. The interactions among these can be find in Fig. 3 [27], [31]. Rot is an another important regulatory locus that was predicted as a global transcriptional repressor of virulence genes, whose activity could be counteracted by RNAIII [35]–[37]. Microarray analysis demonstrated that Rot is a negative regulator of many genes encoding extracellular virulence factors [38], [39].

### TCR Signaling Pathway in *H. sapiens*

T cell receptor (TCR) activation promotes a number of signaling cascades that ultimately determine cell fate through regulating cytokine production, cell survival, proliferation, and differentiation. An early event in TCR activation is phosphorylation of immunoreceptor tyrosine-base activation motifs (ITAMs) on the cytosolic side of the TCR/CD3 complex by lymphocyte protein-tyrosine kinase (Lck). The CD45 receptor tyrosine phosphatase modulates the phosphorylation and activation of Lck and other Src family tyrosine kinases. z-chain associated protein kinase (ZAP70) is recruited to the TCR/CD3 complex where it is activated and phosphorylation of downstream adaptor or scaffold proteins. Phosphorylation of SLP-76 by ZAP70 promotes recruitment of Vav (a guanine nucleotide exchange factor), the adaptor proteins NCK and GADS, and an inducible T cell kinase (ITK) [40]. Phosphorylation of phospholipase C
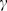
1 (PLC
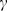
1) by ITK results in the hydrolysis of phosphatidylinositol 4,5-bisphosphate (PIP2) to produce the second messengers diacylglycerol (DAG) and inositol trisphosphate (IP3).

**Figure 4:**
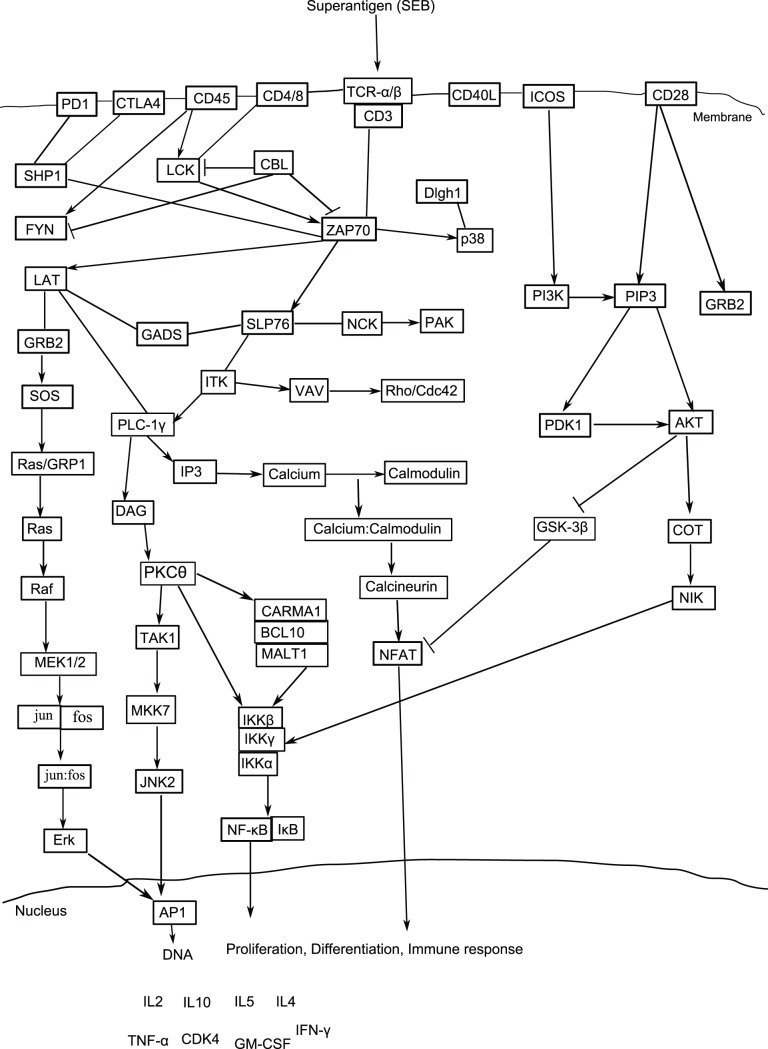
TCR signaling pathway of *H. sapiens*. SEB refers to Staphylococcal Enterotoxin B. Sharp arrow head indicates activation and blocked arrow shows inhibition.

DAG activates PKC-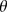 and the MAPK/Erk pathways, both of these promotes NF-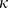
B activation. IP3 triggers the release of Ca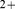 from endoplasmic reticulum (ER), which promotes the entry of extracellular Ca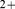 into cells through calcium release-activated Ca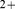 (CRAC) channels. Calcium-bound calmodulin (Ca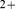/CaM) activates the phosphatase calcineurin, which promotes IL-2 gene transcription through the transcription factor NFAT. Feedback regulation at several points within these pathways allows for different outcomes, depending on the cell type and environment. The incorporation of signals from additional cell surface receptors (such as CD28 or LFA-1) further regulates cellular response. Manually designed TCR signaling pathway has been given in Fig. 4.

### Molecular Interactions between SAg Expression Regulatory Pathway of *S. aureus* and TCR Signaling Pathway of an Infected *H. sapiens*

The binding of SAgs to TCR and/or to MHC class II molecules triggers intracellular biochemical signals for cell activation, differentiation, proliferation and the release of inflammatory cytokines [41]–[44]. In this regard, Seth et al. [45] has provided physical evidence for the existence of SEB-TCR binary complexes and shown that the formation of these complexes increases the binding to MHC II and stabilizes the ternary complex of TCR-SAg-MHC II [45], [46]. A schematic model of interaction between SAg with TCR and MHC class II molecules is shown in Fig. 1.

The interaction of SAgs with MHC class II molecules is important for their ability to stimulate T cells, because accessory cells that do not express class II fail to provide costimulation for the superantigenic response [47]–[50]. Each SAg has a signature specificity for a set of V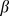 families and can interact with all T cells expressing those V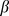 elements regardless of the antigenic specificity of their TCR. Due to this reason, antigens can interact with one T cell in every 10^4^ or 10^6^ T cells, whereas SAgs are capable of interacting with as many as 5 to 20% of resting T cells. It is important to mention that this number can vary depending on the number of V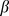 families that recognize a given SAg and on the frequency of T cells expressing those V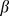 families in each individual’s repertoire. The distinct binding sites for antigen and SAg has been shown in Fig. 1. The other difference is, processed antigen recognition by the TCR is MHC restricted (*i.e.*, only self MHC class II molecules), whereas the response of T cells to SAg is MHC unrestricted and can occur in the presence of either self or foreign MHC class II molecules.

## Results

**Figure 5:**
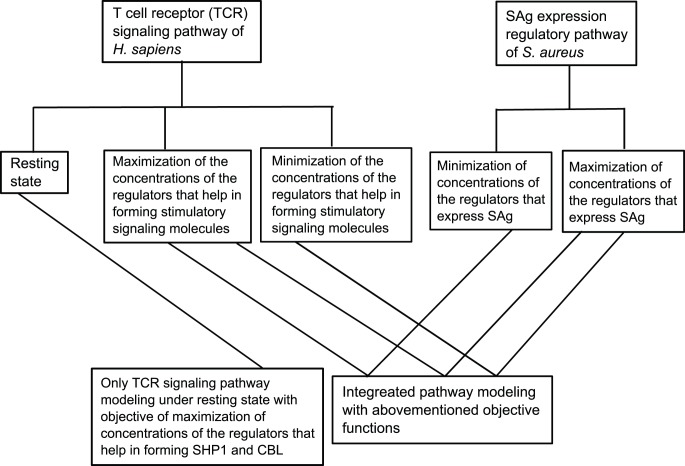
Diagram showing the studied biochemical pathways upon perturbed/unperturbed conditions, along with different conflicting objective functions.

Here, we provide the behavior of the signaling molecules present in TCR pathway of *H. sapiens* in perturbed/unperturbed conditions, with six possible combinations of objective functions for optimization. We have also shown these combinations through a diagram, as given in Fig. 5.

### TCR Signaling Pathway of *H. sapiens* (without Pathogen Perturbation)

First of all, we have implemented the aforesaid method on pathogen unperturbed TCR signaling pathway of *H. sapiens*. We have manually constructed TCR signaling pathway (Fig. 4) from the literature [51]–[54] and as given in different pathway databases, like, KEGG (http://www.genome.jp/kegg/pathway/hsa/hsa04660.html), PANTHER (http://www.pantherdb.org/pathway/pathwayDiagram.jsp?catAccession=P00053), and as given in the websites of companies, CellSignaling Technology (http://www.cellsignal.com/reference/pathway/T_Cell_Receptor.html) and SABiosciences (http://www.sabiosciences.com/pathway.php?sn=TCR_Signaling) among others.

The starting signaling molecule is TCR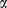/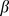. The pathway comprises 64 signaling molecules and 95 interactions. Here, the objective is to increase the quantities of the signaling molecules - CBL and SHP1. They are responsible for keeping TCR signaling pathway in resting state. The importance of SHP1 is evident, as mice deficient in SHP1 develops severe autoimmunity [55]. CBL protein including c-CBL (Cas-Br-M ecotropic retroviral transforming protein) and b-CBL, play an important role as negative regulators of T cell signaling [56]. Here the objective function is 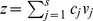, where 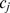 values denote the expression/concentrations of the regulators that maintain the status/concentration of signaling molecules SHP1 and CBL. Similarly, the fluxes, *i.e.*, 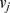 values are the rates of reactions through which SHP1 and CBL form. On maximization of 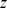, its value has been found to be 1.8732.

### Pathogen Perturbed (Stimulated) TCR Signaling Pathway of *H. sapiens*: Maximization of Stimulatory Molecules in TCR Signaling Pathway

**Figure 6:**
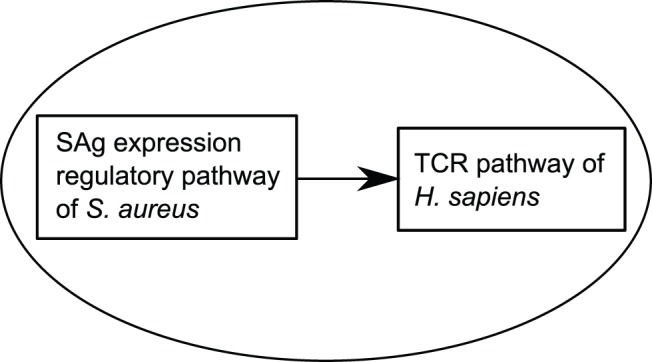
Displaying the concept behind the construction of integrated pathway system that includes SAg expression regulatory pathway of *S. aureus* and TCR signaling pathway of an infected host (*H. sapiens*).

We have constructed the pathogen perturbed integrated pathway comprising aforesaid SAg expression regulatory pathway of *S. aureus* and TCR signaling pathway of *H. sapiens* and is conceptually depicted by Fig. 6. The integrated pathway consists of 99 molecules and 161 interactions. The starting gene is AgrD. Signal transduction through TCR is initiated by sequential activation of PTKs, *viz*., LCK, FYN and ZAP70 [57], the concentrations of which need to be maximized. Thus the objective function is 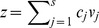. The term 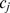
’s signify the expression/concentrations of the regulators that maintain the status/concentration TCR:CD3 complex, ZAP70, LCK and FYN are at their maximum level. Similarly, the fluxes 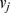
’s are the rates of the reactions through which TCR:CDR3 complex, ZAP70, LCK and FYN form. The value of 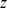 has found to be 10.1155. We have applied the method to demonstrate how toxin expression is regulated in the pathogen, and its effect on the TCR signaling pathway of an infected host.

### Pathogen Perturbed TCR Signaling Pathway of *H. sapiens*: Maximization of SAg Expression in *S. aureus* as well as the Concentrations of Stimulatory Molecules Present in TCR Signaling Pathway

We have chosen this combination of objective function to demonstrate the effect of maximum perturbation on the host’s immune system, when it functions at its best. In this case, we have maximized the objective function in the form of maximizing expression of SAg and the concentration of stimulatory molecules of TCR signaling pathway present in the abovementioned integrated pathway. For this purpose, the objective function is 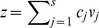, where 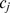
’s are the concentrations of positive regulators that is required for the expression of toxin and for the regulators that maintain the status/concentration of TCR:CDR3 complex, ZAP70, FYN and LCK signaling molecules. Similarly, 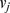
’s signify the fluxes through which toxin molecule and TCR:CDR3, ZAP70, FYN and LCK form in an integrated pathway system. The value for 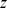 has been computed as 15.0036. The resulting concentrations of the regulators responsible for forming signaling molecules present in TCR signaling pathway is given in Table 1 (column 4).

**Table 1:** Names of signaling molecules present in TCR signaling pathway of *H. sapiens* and the respective *c*-values for the regulators to form these signaling molecules in unperturbed/perturbed conditions in different cases as mentioned in Fig. 5.

7*Signaling molecules	UnperturbedTCR pathway	PathogenperturbedTCR pathway	PerturbedTCR pathway(maximization oftoxin expressionand of TCRstimulatorymolecules)	Perturbed TCRpathway(conflictingobjectives’optimization)	Maximization ofSAg expressionand minimizationof TCR stimulatorymolecules	7*Remarks
SHP1	0.67791	0.3227	0.4614	0.34291	0.93821	justified
CBL	0.99269	0.5974	0.12434	0.21257	1	justified
LCK	0.026283	0.91611	0.93833	0.39188	0.1767	justified
FYN	0.40972	0.86331	0.64034	0.76781	0	justified
ZAP70	0.30989	0.48601	0.74887	0.41506	0	justified
p38	0.44236	0.72841	0.98794	0.32203	0.005517	justified
SLP-76	0.051128	0.60307	0.58992	0.5495	0.29318	justified
LAT	0.25764	0.57508	0.50586	0.15188	0.60794	justified
VAV	0.24826	0.7427	0.84067	0.53051	0.19772	justified
Rho/Cdc42	0.5369	0.57143	0.41254	0.31557	0.34745	justified
Raf	0.42317	0.49728	0.72518	0.44493	0.032599	justified
PLC*γ*1	0.39187	0.47768	0.46287	0.4012	0.083833	case (3) is not justified
IP3	0.62567	0.77416	0.19291	0.92249	0.79828	cases (3) and (4) are not justified
*Ca* ^2+^	0.20847	0.73472	0.45354	0.30107	0.07147	cases (4) and (5) are not justified
PKC*θ*	0.77424	0.85534	0.9583	0.15222	0.043483	justified
MEK1/2	0.11708	0.12904	0.008478	0.33124	0.36072	case (3) is not justified
Erk	0.14316	0.17367	0.79652	0.589	0	case (5) is not justified
JNK2	0.53459	0.61927	0.71362	0.68198	0.41653	justified
AP1	0.63463	1	0.65477	0.81176	0.94164	cases (4) and (5) are not justified
NCK	0.654	0.67508	0.50586	0.042724	0.60794	case (5) is not justified
PAK	0.17261	0.20377	0.51681	0.096005	0.055632	justified
CARMA1:BCL10:MALT1	0.39768	0.81185	0.45733	0.23229	0.072425	case (3) is not justified
IKK*β*:IKK*γ*:IKK*α*	0.70419	0.90608	0.35092	0.71188	0.36092	cases (3) and (5) are not justified
NF-*κ*B	0	0.2298	0.92031	0.19048	0.55139	justified
PI3K	0.38225	0.75573	0.46976	0.71432	0	case (5) is not justified
AKT	0.032244	0.95152	0.59375	0.093664	0.82004	justified
COT	0.21357	0.35455	0.41137	0.33215	0.42996	case (3) is not justified
GSK-3*β*	0.16808	0.17778	0.25034	0.17768	0.73146	case (3) is not justified
GRB2	0.64977	0.81376	0.80354	0.18871	0.25285	justified
IL-2	0.83085	0.84865	0.05107	0.81404	0.82636	cases (3) and (4) are not justified
IL-4	0.95409	0.23093	0.56354	0.14729	0.85952	justified
IL-5	0.44216	0.23857	0.14132	0.15953	0.94388	case (3) is not justified
IL-10	0.16035	0.62864	0.60184	0.96351	0.6764	case (4) is not justified
IFN-*γ*	0.15738	0.80446	0.19918	0.092446	0.38047	case (3) is not justified
TNF-*α*	0.23533	0.25161	0.12094	0.14676	0.76533	case (3) is not justified
CDK-4	0.062237	0.16392	0.40135	0.45356	0.14987	case (5) is not justified

The term ‘justified’ in the last column (Remarks) indicates that results obtained by the present method are in accordance to the general biological context.

### Pathogen Perturbed TCR Signaling Pathway of *H. sapiens*: Optimization of Conflicting Objective Functions (Minimization of SAg Expression in *S. aureus* and Maximization of the Concentrations of the Stimulatory Molecules in TCR Signaling Pathway in *H. sapiens*)

Next, on the same integrated pathway, we have opted to optimize two conflicting objectives simultaneously, *i.e.*, minimization of toxin expression in a pathogen and maximization of the status/concentrations of TCR:CD3 complex and ZAP70 in a TCR signaling pathway of the infected host. Thus the objective function can be expressed as 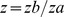, where 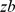 is the objective function, which is to be minimized. Here 
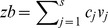
, 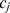
’s refer to the concentrations of SAg and 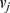
’s signify the flux for the expression of toxin in a pathogen. Similarly, 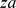 requires to be maximized, where 
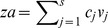
. Here 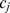
’s refer to the expression/concentrations of the regulators that maintain the status/concentration TCR:CD3 complex and ZAP70. Similarly, 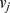
’s refer to the rate of reaction at which TCR:CD3 complex and ZAP70 are formed/maintained, respectively.

**Figure 7:**
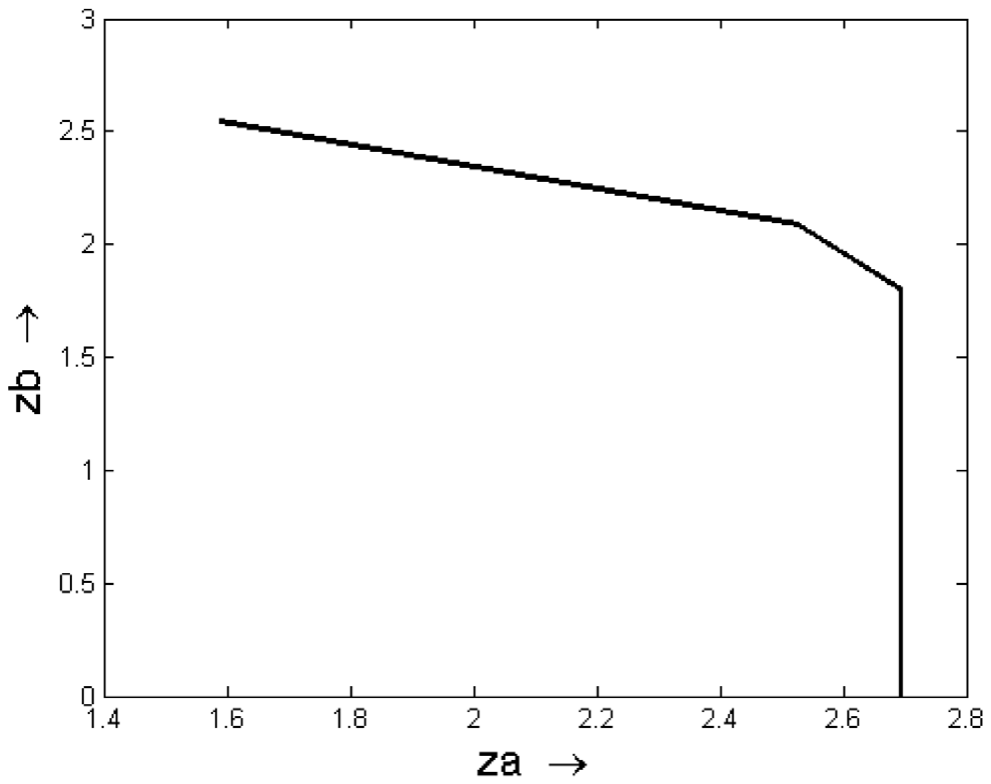
Graph shows optimization of two conflicting objective functions in an integrated pathway system of SAg expression regulatory pathway of *S. aureus* and TCR signaling pathway of an infected host (*H. sapiens*). Here, 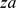 is maximized, while 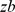 is minimized.

**Figure 8:**
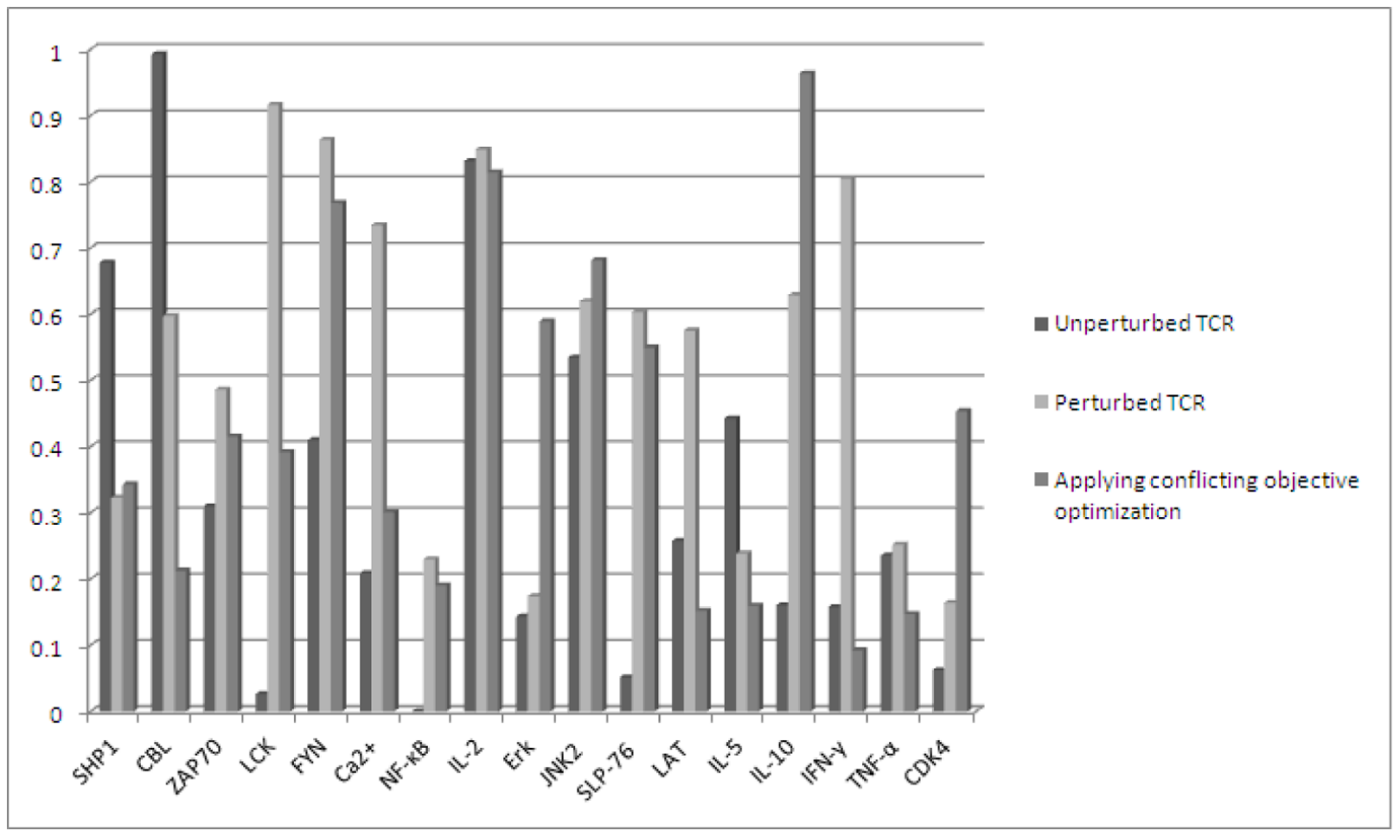
Comparing 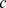-values [0–1] of the molecules present in TCR signaling pathway of *H. sapiens* in three cases: (1) in an unperturbed TCR signaling pathway, (2) in a perturbed one and (3) after applying conflicting objective function optimization (case 4) on the perturbed TCR signaling pathway.

Here, we have aimed at finding out whether minimization of the toxin production in a pathogen leads to the change in the concentrations of the proteins coded by TCR signaling pathway genes in the infected host. The optimization of two conflicting objective functions is shown in Fig. 7. We have analyzed the concentrations (*c*-values) of regulators quantitatively, through which these TCR signaling pathway proteins form. In this case, we have found the lower (in comparison to the *c*-values obtained for case (2)) expression/concentrations of regulators that maintain the concentrations of the defense molecules (*e.g.*, ZAP70, LCK and FYN) present in TCR signaling pathway of *H. sapiens*. It is due to toxin minimization in *S. aureus* in comparison to pathogen perturbed TCR signaling pathway in which SAg expression was kept unperturbed (Fig. 8). However, in this case, these values are higher for the regulators of ZAP70, LCK and FYN than the values obtained for them in the case (1), where there is no pathogen attack upon host. The comparison among the *c*-values for the aforesaid conditions is given in Table 1. The variation in *c*-values for this case is justified for most of the TCR signaling molecules as mentioned in column 7 (Remarks) of Table 1. We have found that minimized toxin expression in a pathogen has a lesser effect on the stimulation of host’s TCR signaling pathway at the time of attack than that with higher toxin expression. This phenomenon has been explained in column 7 (Remarks) of Table 1.

### Pathogen Perturbed TCR Signaling Pathway of *H. sapiens*: Optimization of Conflicting Objectives (Reverse of the above Case - Maximization of SAg Expression in *S. aureus* and Minimization of the Concentrations of Stimulatory Molecules in TCR Signaling Pathway in *H. sapiens*)

Here we would like to observe the behavior of the host immune system at the time of maximum perturbation, when the host immune system is at its weakest status. For this purpose, we have chosen the same integrated pathway as above, with 99 molecules and 161 interactions. The objective function is 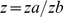, where 
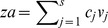
 is for maximization. Here the term 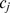
’s denote the concentrations of positive regulators that leads to the expression of a toxin molecule in a pathogen and 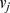
’s refer to the flux rate through which toxin molecule forms. Similarly, 
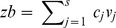
 is for minimization, where term 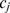
’s refer to the concentrations of regulators that maintain the expression/concentration of TCR:CD3 complex and ZAP70. Similarly 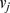
’s refer to rate of reaction at which TCR:CD3 complex and ZAP70 are formed/maintained. The value 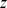 has been computed to 0.0944. The *c*-values for different combinations of conflicting objective function optimization have been given in Table 1 (see the column 6), where they can be compared with the others.

Column 7 (Remarks) of Table 1 indicates that whether the changes in *c*-values obtained for 6 different cases are justified. The biological significance of this variation in *c*-values lies in the choice of objective functions during optimization in 6 different cases. As we have mentioned earlier, we have found higher concentrations of regulators that maintain the concentrations of TCR signaling inhibitory molecules (SHP1 and CBL) in resting state of the T cells (no pathogen attack, case (1)). However, in the same case, the concentrations of regulators that maintain the concentrations of TCR signaling stimulatory molecules (*e.g.*, ZAP70, LCK, FYN) would be lower. The reason behind this variation is that TCR signaling pathway does not need to be stimulated as there is no pathogen perturbation upon host. Likewise, we have considered six cases and almost all the TCR signaling molecules for analysis in these cases. From column 7 (Remarks) of Table 1, it is clear that the variations of *c*-values obtained for most of the cases are justified in biological context.

### Perturbation of the Signaling Molecules Responsible for Stimulation of TCR Signaling Pathway upon Pathogen Attack: Generation of a Hypothesis

The understanding of the control and regulation of TCR signaling is a key point to explore an adaptive immune system. It is mentioned earlier that activation of SYK family kinase (ZAP70) is an essential step in TCR signaling, which later phosphorylates the protein adaptor molecule, Linker for Activation of T cells (LAT). There exist two more protein tyrosine phosphate (PTKs), *viz.*, LCK and FYN, which initiates TCR signals. The regulation of LCK is critical for maintaining the resting state of the T cell and for signal transduction that includes tyrosine phosphoprotein induction as shown in LCK deficient studies [58], [59].

Taking these evidences of ZAP70, LCK and FYN into considerations, we have chosen the perturbation of their signals to check the effect on the signaling for inhibitory molecules (SHP1 and CBL) as well as on immune response cytokines. SHP1, a tyrosine phosphatase, regulates the level of active tyrosine kinases in peripheral T cells [30] as it is involved in negative feedback loop. b-CBL acts downstream of the TCR and CD28, negatively regulates the phosphorylation of VAV1 and targets the activated TCR:CD3 complex and associated ZAP70 [60].

We have perturbed the concentrations of the signaling molecules, *viz.*, ZAP70, LCK and FYN, which are responsible for stimulating the TCR signaling pathway upon infection. These signaling molecules are otherwise kept as inhibited by signaling molecules, CBL and SHP1. We have performed it upon two different conditions, *viz.*, without and with pathogen perturbation, along with different objective functions. 1) We have opted for minimization of the expression/concentrations of the aforesaid signaling molecules, in an unperturbed TCR signaling pathway and 2) maximization of the same in a perturbed one. For these objective functions, we have observed the changes in concentrations of regulators for the reactions through which CBL and SHP1 form, and for immune response molecules, like, IL-2,-4,-5,-10, IFN-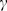, TNF-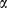 and CDK4. Here, we have found higher *c*-values for CBL and SHP1 in an unperturbed (resting) TCR signaling pathway, for which we have minimized the concentrations of ZAP70, LCK and FYN. In contrast to this, for perturbed (stimulated) TCR signaling pathway, where we have maximized the concentrations of the abovementioned signaling molecules, we have found lower *c*-values for CBL and SHP1 (due to pathogen infection). Moreover, we have also observed *c*-values for above mentioned immune response molecules in both the aforesaid cases. *c*-values for most of the immune response molecules were lower in an unperturbed TCR signaling pathway, where concentrations were minimized for ZAP70, LCK and FYN as an objective function, while it was higher in the other case as mentioned in Table 2.

**Table 2:** Effects on some of the TCR signaling molecules upon perturbing the concentrations of ZAP70, LCK and FYN for unperturbed and perturbed TCR signaling pathways of *H. sapiens*.

Signaling molecules	*c*-values in an unperturbed TCR pathway(upon minimization of concentrationsof the regulators for forming ZAP70,LCK and FYN)	*c*-values in a perturbed TCR pathway (upon maximization of concentrations of the regulators for forming ZAP70, LCK and FYN)
SHP1	0.69453	0.24715
CBL	0.791 8	0.52688
IL-2	0.72529	0.19636
IL-4	0.11492	0.69064
IL-5	0.0076967	0.8236
IL-10	0.99206	0.4812
IFN-*γ*	0.96845	0.059268
TNF-*α*	0.85123	0.62606
CDK4	0.017747	0.35755

Thus we can hypothesize a relation between resting and stimulatory states, and for the behavior of the responsible signaling and immune response molecules in TCR signaling pathway. This hypothesis is based on different *c*-values obtained for chosen signaling molecules upon resting and perturbed conditions, along with the choice of different objective functions. The hypothesis is as follows. The expression/concentrations of regulators that maintain the status/concentrations of the inhibitory molecules will be decreased upon pathogen attack on the host system. In contrast, the expression/concentrations of regulators that maintain the status/concentrations of the stimulatory molecules will be increased at this condition. These activities altogether leads to the stimulation of host immune system at the time of pathogen attack. Moreover, we would also like to mention that the concentrations of regulators that maintain the concentrations of the inhibitory molecules would be higher in resting TCR signaling system (with no pathogen perturbation). This is due to the fact that TCR signaling system does not require to be stimulated if there is no perturbation upon host. However, it would be lower upon pathogen attack on host as now the TCR signaling system requires to be activated/stimulated in order to fight against the pathogen.

### Biological validation of the results

In this Section, we provide the biological validation of the results obtained from abovementioned methodology implemented on six different cases. Here, we compare our results with existing experimental evidences on TCR signaling pathway following the stimulation with Staphylococcal enterotoxins. We have mostly compared the *c*-values under unperturbed, pathogen perturbed and perturbation with conflicting objectives (case 4) in the following text. The abstract of this Section has been given in Table 3. In this table, we have cited the references with related text information to validate the computed values of some of the molecules of TCR signaling pathway of *H. sapiens* for a particular perturbation condition.

**Table 3:** Comparison of some of the computed *c*-values for given signaling molecules with the existing experimental evidences, for biological validation purpose.

Signaling molecules	*c*-values (computed) ina particular condition	Related experimental evidences
NFAT	0.22772 (un)	b-CBL mediated ubiquitination and proteasomal degradation of PLC-1 and PKC that results in decreased NFAT activation [141].
PKC-*θ*	0.85534 (per)	It has been found that calcineurin and PKC-*θ* specifically synergizes to induce transcription of reporters driven by c-jun and IL-2 promoters [74].
PLC-*γ*	0.39187 (un)	b-CBL inhibits activation of the p85 subunit of phosphoinositide 3-kinase (PI3K) [71], PKC-*θ* and phospholipase C-1 (PLC-1) [72], [73].
Ca^2+^	0.73472 (per)	Even without crosslinking, SEB activates protein tyrosine kinase and phosphoinositide-specific phospholipase C that cleaves phosphoinositide into DAG and IP3 which in turn, releases free Ca from endoplasmic reticulum (ER) [75].
IP3	0.77416 (per)	It has been found that initial response of calcium mobilization depends on the release of *Ca* ^2+^ from intracellular store in an inositol-triphosphate (IP3)-dependent pathway [83].
LCK	0.91611 (per)	TCR stimulated control cells exhibited an increase in the phosphorylation of LCK activating Tyr394 [93].
SLP-76	0.60307 (per)	TCR-induced PI3K/Akt activation not only needs membrane translocation and tyrosine phosphorylation of SLP-76, but also LAT for the localization of SLP-76 to the membrane. [97].
LAT	0.57508 (per)	A critical role for both LAT and SLP-76 has been demonstrated by successfully reconstituting LAT-deficient Jurkat T cell lines with LAT or SLP-76 deliberately targeted to the rafts [65], [98].
VAV	0.7427 (per)	Disruption of TCR signaling pathway in VAV-deficient mice had shown decrease in interleukin-2 (IL-2) secretion and TCR-mediated cytoskeletal reorganization in T cells [130], [131].
Dlg1	0.99428 (un)	Dlg1 localizes with actin cytoskeleton in T cells and associates with early participant molecules of the signaling process. It functions as a negative regulator of T cell activation [103].
NCK	0.67508 (per)	NCK, a ubiquitously expressed adapter protein, plays a pivotal role in TCR-induced reorganization of the actin cytoskeleton and the formation of the immunological synapse in T lymphocytes [123].
PAK1	0.017261 (un)	PAK1 belongs to a family of closely related serine/threonine kinases and its activation contributes to TCR-induced ERK activation, calcium flux and the NFAT transcriptional response [125], [126].

Here, ‘un’ denotes unperturbed TCR signaling pathway and ‘per’ refers to perturbed conditions with conflicting objective function optimization.

### Effect on Molecules of TCR Signaling Pathway of Unperturbed and Pathogen Perturbed Host Systems: A Comparison

As mentioned earlier, we have studied and compared the behavior of TCR signaling pathway for six different cases:

1. First of all, we have implemented the method over an unperturbed (resting state) TCR signaling pathway of *H. sapiens* and observed *c*-values of the signaling molecules.
2. Pathogen perturbed (stimulated) TCR signaling pathway has been investigated for which SAg expression regulatory pathway of *S. aureus* and TCR signaling pathway of *H. sapiens* has been integrated.
3. We have maximized both SAg expression in *S. aureus* as well as the concentrations of the stimulatory molecules (ZAP70, LCK and FYN) to study the behavior of perturbed TCR signaling pathway molecules.
4. We have studied the behavior of the same integrated pathway over the optimization of two conflicting objective functions for which, we have applied a conflicting objective function optimization under FBA and compared the *c*-values. Here, minimization of SAg expression and maximization of the concentrations of stimulatory molecules (TCR:CD3 complex and ZAP70) have been done.
5. We have explored the behavior of the same integrated pathway, upon optimization of two conflicting objective functions, however, here the conflicting objectives are just opposite of the case (4).
6. We have also compared the *c*-values for CBL, SHP1 and interleukins, while perturbing the concentrations of the some of the signaling molecules, *viz.*, ZAP70, LCK and FYN, under pathogen perturbed and unperturbed conditions.

We have assumed higher *c*-values in [0–1] for inhibitor molecules, like, for CBL and SHP1, in the absence of any pathogen attack. In the presence of a pathogen (pathogen perturbed system), we have assumed higher *c*-values for the stimulatory molecules, like, ZAP70, LCK among others, which later produce cytokines and interleukins. The fourth case is of optimization of conflicting objective functions, where we have assumed comparatively lower *c*-values of stimulatory molecules due to toxin minimization in *S. aureus*. On the basis of above assumptions, we have compared *c*-values and validated the results through existing experimental observations for TCR signaling pathway stimulated by Staphylococcal enterotoxins (direct evidences) or otherwise (indirect evidences). The comparison of *c*-values for some of the signaling molecules based on abovementioned three cases are given in Fig. 8. On the basis of analyzing different cases, we have hypothesized that perturbation (minimization or maximization of the concentrations of the chosen molecules) of TCR signal stimulatory molecules, under different conditions, have an impact on overall TCR signaling in the form of varied signaling patterns of inhibitory TCR signaling and immune response molecules.

There are some negative regulators for TCR signaling pathway that inhibit TCR-mediated downstream signaling, *e.g.*, E3 ubiquitin ligases c-CBL, b-CBL, SHP1 among others [30], [61]–[63]. There are certain molecules, like, CTLA4 (cytotoxic T lymphocyte associated antigen 4) and PD1 (programmed cell death 1), which act as another layer of feedback regulation of TCR signaling. At the molecular level, b-CBL acts at the downstream of the TCR and CD28 by negatively regulating phosphorylation of the guanine nucleotide exchange factor VAV1 and thus impacts lipid raft aggregation apart from targeting the activated TCR-CD3 complex and associated ZAP70. In addition, b-CBL mediates ubiquitination and proteasomal degradation of PLC
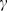
1 and PKC that results in decreased NFAT activation. This is the reason behind getting lower (0.022772) *c*-value for NFAT in an unperturbed TCR signaling pathway, whereas, it is increased (0.51727) due to the effect of SAg on TCR signaling. In the third case, it has become 0.30107 on SAg expression minimization.

It has also been found that c-CBL predominantly acts as a negative regulator in the thymus and b-CBL negatively regulates mature CD28-dependent T cell activation [64]. We have founded higher *c*-value for signaling molecule CBL in an unperturbed system that further inhibit LCK, FYN and ZAP70. We have got *c*-value of 0.99269 for CBL in pathogen unperturbed system, whereas it has become 0.5974 and 0.21257 for pathogen perturbed system and for the system, where conflicting objectives have been optimized. It is the same case with SHP1, which inhibits ZAP70 and TCR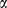:CD3 complex. Tyrosine phosphorylation and dephosphorylation of proteins play a critical role for many T cell functions and thus it is now accepted that PTKs are essential during T cell signaling. SHP1 is one of the two members of Src-homology 2 domain (SH2)-containing PTPs [65]. SHP1 has been implicated in negative regulation of signaling events induced by receptors for antigens, cytokines and growth factors [30], [66]. Over-expression of SHP1 in T cell lines leads to inhibition of TCR mediated phosphorylation of TCR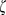 chain, association of Zap70 with TCR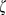 chain, phosphorylation of LAT and IL-2 production [67], [68]. These data suggest a model, in which SHP1 is a negative regulator of TCR-mediated signaling in T cells. The expression of catalytically active mutants of SHP1 had not shown any detectable effect on TCR-mediated signaling while expression of full length SHP1 inhibits TCR-mediated signaling, as evidenced by inhibition of IL-2 production [68]. These experimental observations for SHP1 show the similarity with our computed *c*-values. The *c*-value for the regulators of SHP1 is higher (0.67791) in a pathogen unperturbed pathway, where as it has become 0.3227 at the time of pathogen attack and 0.34291 during conflicting objective function optimization (refer Table 1).

The intracellular signals underlying programmed cell death suggests that DAG activated PKC may serve as a negative regulator. Lin et al. [69] have found the level of protein phosphorylation was reduced in thymocytes after SEB treatment for 24 hours. The activity of classical PKC subspecies were found to be weakened in both cytosolic and membrane fractions of thymocytes. It was shown that concomitant loss of b-CBL restored the defective proliferation and IL-2 secretion responses of PKC-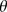 deficient T cells induced by either anti-CD3 and anti-CD28 or by SEB back in comparison of wild type cells [70]. Moreover, b-CBL inhibits activation of the p85 subunit of PI3K [71], PKC-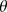 and PLC-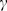
1 [72], [73]. It also acts with c-CBL and promotes antigen-induced down-regulation of TCR [56]. We have also got similar results in the form of *c*-values, *i.e.*, lower value (0.77424) for PKC-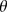 in an unperturbed TCR signaling pathway. In contrast, *c*-values has become 0.85534 and 0.15222 in a pathogen perturbed system and in the system, where it is optimized for two conflicting objectives, respectively. PKC-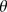 specifically cooperates with calcineurin and their signals converge on Rac that leads to JNK activation. Similarly, it has been found that calcineurin and PKC-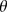 specifically synergizes to induce transcription of reporters driven by c-jun and IL-2 promoters [74]. It is in the same manner for PLC-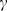
1, *i.e.*, *c*-value of 0.39187 for PLC-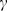
1 in an unperturbed TCR signaling pathway. In contrast, it has become 0.47768 and 0.4012 for a pathogen perturbed system and in the system, where it is optimized for two conflicting objective functions, respectively.

SEB mobilizes intracellular free calcium (Ca) in HLA-DR positive but not in HLA-DR negative keratinocytes [75]. Without crosslinking, SEB can activate PTK and PLC that cleaves phosphoinositide into DAG and IP3 which, in turn, releases free Ca from endoplasmic reticulum (ER). This is the reason, we have got lower *c*-value of 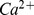 (0.20847) in an unperturbed TCR signaling pathway, whereas, it has increased (0.73472) due to the effect of SAg on TCR signaling. In the third case, it has become 0.30107 on SAg expression minimization. It is well known that costimulation of TCR and CD28 is required for optimal interleukin-2 (IL-2) induction. These signals, which can be replaced by the pharmacological agents phorbol ester (PMA) and 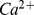 ionophore, synergistically activate JNK [76]. Even Staphylococcus Enterotoxin A (SEA) has shown to induce an increase in intracellular 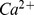 in human intestinal epithelial cells [77]. Calcineurin plays a major role in signal transduction leading to T cell activation [78]. Calcineurin dephosphorylates NFATc proteins [79], [80] and induces IL-2 transcription in cooperation with PKC [81], [82]. It has also been found that initial response of Ca mobilization depends on the release of 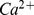 from intracellular store in an inositol-triphosphate (IP3)-dependent pathway [83]. We have found lower (0.62567) *c*-value for IP3 in an unperturbed TCR signaling pathway. In contrast, it has become 0.77416 and 0.92249 in a pathogen perturbed system and in the system, where it is optimized for two conflicting objective functions, respectively.

A study has shown to block TNF-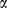 induction in SEB induced PKC translocation, and pretreatment of cultures with inhibitors of PKC [84]. Alteration of levels of diacylglycerol (DAG) by treatment with inhibitors of phospholipase C (PLC) or DAG kinase altered SEB-induced TNF-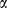 production. This information suggests that PKC activation plays a critical role in SEB-induced TNF-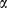 production in human T cells. Moreover, there are several studies that show induction of TNF-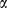 by SEA/SEB/TSS1 [85], [86]. Here we have given *c*-value for both DAG and PKC-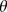. We have found lower (0.68837) *c*-value for DAG in an unperturbed TCR signaling pathway. On the other hand, it has become 1 in a pathogen perturbed system and in the system, where it is optimized for two conflicting objective functions, respectively. For PKC-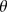, it is 0.77424 in an unperturbed TCR signaling pathway, whereas, it has become 0.85534 and 0.15222 in a pathogen perturbed system and in the system, where it is optimized for two conflicting objective functions.

It is well known that the activation of the src-family kinases LCK and FYN is central to the initiation of TCR signaling pathways [87]. A study has shown a requirement for the LCK-SH3 domain in optimal T cell development [88]. Biochemical studies have indicated that LCK plays a key role in initiating TCR signals through receptor phosphorylation and activation of ZAP70 tyrosine kinase [89], [90]. CD45 tyrosine phosphatase maintains the inhibitory C-terminal residues of LCK and FYN in a dephosphorylated form that keeps basally active conformation of these proteins [91], [92]. A study by Dong et al. [93] indicates that a substantial pool of LCK and FYN is active in naive CD4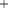 T cells. TCR stimulated control cells showed an increase in the phosphorylation of LCK activating Tyr394 (p56 and p59). The authors have also shown that LAT depletion reduces FYN phosphorylation upon TCR stimulation by SEB. Thus LAT promotes TCR-induced phosphorylation of LCK and FYN tyrosines. Here we can show the similarity between the existing experimental observations and the computed *c*-values. We have found lower (0.026283) *c*-value for LCK in an unperturbed TCR signaling pathway. In contrast, it has become 0.91611 and 0.39188 in a pathogen perturbed system and in the system, where it is optimized for two conflicting objective functions, respectively. In the same way, we got lower (0.40972) *c*-value for FYN in an unperturbed TCR signaling pathway. In contrast, it has become 0.86331 and 0.76781 in a pathogen perturbed system and in the system, where it is optimized for two conflicting objective functions, respectively.

Li et al. [94] have investigated the activation of signaling molecules present at upstream of ERK and downstream of TCR in W97ALck expressing cells, and reported that the inability of W97ALck to support ERK activation was due to defect in the activation of Raf-1. One more study [95] has reported that signaling ERK was dependent on the activation of Golgi membrane-associated N-Ras. Moreover, basal and stimulated levels of Erk-type MAPK phosphorylation were found to be sensitive to Mek1 inhibitor PD-98059 that indicates that the bacterial products activated the entire signaling cascade that coregulates IL-8 induction and secretion [96]. We have also found lower (0.40366) *c*-value for RasGRP1 in an unperturbed TCR signaling pathway. In contrast, it has become 0.58313 and 0.51328 in a pathogen perturbed system and in the system, where it is optimized for two conflicting objective functions, respectively. Moreover, we have got lower (0.11708) *c*-value for MEK1/2 in an unperturbed TCR signaling pathway, respectively. In contrast, it has become 0.12904 and 0.33124 in a pathogen perturbed system and in the system, where it is optimized for two conflicting objective functions respectively. We have found lower (0.42317) *c*-value for Raf in an unperturbed TCR signaling pathway. In contrast, it has become 0.49728 and 0.44493 in a pathogen perturbed system and in the system, where it is optimized for two conflicting objective functions, respectively.

Shim et al. [97] have described the role of SLP-76 and LAT in the activation of PI3K signaling due to TCR stimulation. Their results showed that TCR-induced PI3K/Akt activation needs both membrane translocation and tyrosine phosphorylation of SLP-76 as well as LAT for the localization of SLP-76 to the membrane. A critical role for both LAT and SLP-76 has been demonstrated through reconstitution of LAT-deficient Jurkat T cell lines with LAT or SLP-76 deliberately targeted to the rafts [65], [98], [99]. We have found lower (0.051128) *c*-value for SLP-76 in an unperturbed TCR signaling pathway. In contrast, it has became 0.60307 and 0.5495 in pathogen perturbed system and in the system, where it is optimized for two conflicting objective functions, respectively. In the same manner, we have found lower (0.25764) *c*-value for LAT in an unperturbed TCR signaling pathway. In contrast, it has become 0.57508 and 0.15188 in pathogen perturbed system and in the system, where it is optimized for two conflicting objective functions, respectively. In LAT-deficient J.Cam2 [100] and ANJ3 cells [98], PLC
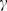
1, VAV and SLP76 phosphorylation were not detected and TCR-mediated signaling events were found to be impaired. Early T cell development has been found to be blocked in LAT-negative mice similar to that observed in mice, in which either LCK and FYN, or ZAP70 and Syk was deleted [101]. These observations demonstrate that LAT is an obligatory step, downstream of LCK and ZAP70, and is an important link to Ras/MAP kinase and PLC
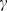
1 pathways.

Dlg1 is expressed in all T cell developmental stages and forms a stable complex with LCK [102]. It functions as a negative regulator of T cell activation. Endogenous Dlg1 connects with TCR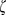 and CBL [103]. The transient overexpression of Dlg1 attenuates basal and VAV1-induced NFAT reporter activation [103]. It is also shown that the reduction of Dlg1 expression by RNA interference enhances both CD3- and SAg-mediated NFAT activation. Thus it is interpreted that Dlg1 acts as an activation antagonist. This is the reason, we have got higher (0.99428) *c*-value for Dlgh1 (or Dlg1) in an unperturbed TCR signaling pathway. In contrast, it has become 0 and 0.46094 in pathogen perturbed system and in the system, where it is optimized for two conflicting objective functions, respectively.

The cytoskeleton of eukaryotic cells has a pivotal role in host-pathogen interactions as it is essential for epithelial and endothelial barrier functions. It limits the invasion and dissemination of pathogens in tissues [104]. GTP-binding proteins of the Rho family are regulators of the actin cytoskeleton [105], and Rho proteins play essential roles in host cell invasion by bacteria. They are the targets for bacterial protein toxins that either inactivate GTPases by ADP-ribosylation or glucosylation, or activate them by deamidation. Specifically, Rho GTPase Cdc42 regulates cytoskeletal changes at the immunological synapse that are critical to T cell activation [106], [107]. Evidence suggests that actin assembly at the cell-cell interaction site is nucleated by the Arp2/3 complex and/or formins, following activation by Rho GTPases such as Cdc42 and Rac1. We have found higher (0.53691) *c*-value for Rho/Cdc42 in an unperturbed TCR signaling pathway. In contrast, it has become 0 and 0.31557 in a pathogen perturbed system and in the system, where it is optimized for two conflicting objective functions, respectively.

### Effect on Immune Response Signaling Molecules

SAgs have been demonstrated to act as potent inducers of several proinflammatory cytokines in the APCs such as interleukin-1 (IL-1) and tumor necrosis factor-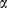 (TNF-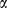). Staphylococcal toxins induce the production of interleukin-1 (IL-1) and tumor necrosis factor-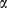 (TNF-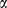) in human monocytes [108]. In keratinocytes, TNF-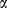 production is enhanced by staphylococcal toxins [109] and in lymphocytes they induce IL-1, IL-2, IL-4, IL-6, IL-10, TNF-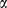 and IFN-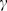 production [110]. We have got lower (0.83085) *c*-value for IL-2, which has become higher (0.84865) at the time of infection, and has again been lower (0.83404) when we optimized the integrated pathway system for two conflicting objective functions. The case is similar to IL-10, IFN-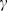, TNF-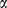 and CDK4 (*c*-values can be compared as given in Table 1). It has been shown that SE induces changes in the expression and signal transduction through IL-2 receptor (IL-2R) 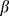 and 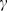 chains (IL-2R 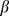 and IL-2R 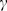) in human CD4+ T cell lines [111]. Here the expression of IL-2R 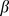 was down-regulated, IL-2R 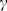 was slightly up-regulated, while IL-2R 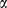 remained largely unaffected due to 4 hours of exposure to SEA and SEB. Gene expression profile showed up-regulation of genes involved in several proinflammatory pathways after 3 hours post-intranasal challenge with SEB in HLA class II transgenic mice, along with several hundred-fold increase in serum levels of pro-inflammatory cytokines, like, TNF-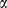 and IFN-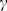 [112]. Excessive interferon gamma (IFN-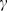) release due to SEB has been linked with the development of a life-threatening systemic inflammation [113]. We can find its similarity with our results in the form of higher (0.80446) *c*-value for IFN-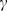 in an infected TCR signaling pathway.

It should be mentioned here that repeated injections of enterotoxin in mice resulted in high levels of IL-10, but there was also a dramatic decrease in all the other cytokines (IL-2, IL-4, TNF-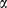, IFN-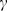) [114]. A study by Ackermann et al. [115] in human leukaemic mast cells demonstrated that SAg stimulation downregulates the production of IL-4. Thus, through these findings we can say that SAgs may also downregulate cytokine production.

CARMA1 recruits signaling components, *viz.*, Bcl10 (B-cell lymphoma 10), MALT1 (mucosa-associated lymphoid tissue lymphoma translocation protein 1), and IKK complex into an immunological synapse [116], [117]. Bcl10 and CARMA1 are two adaptor/scaffold proteins, mediates TCR-induced NF-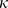
B activation, along with MALT1 (a caspase-like protein that binds to Bcl10). First considering CARMA1, it has been found that oligomerization of CARMA1 is required for TCR-induced NF-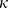
B activation [118]. NF-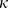
B remains inactive through sequestration in the cytoplasm by inhibitory I
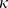
B proteins in quiescent T cells. TCR and CD28 stimulation induces the rapid phosphorylation and ubiquitin-dependent degradation of I
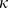
B, leading to translocation of NF-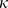
B to the nucleus. PI3K/PKB signaling enhances TCR and CD28-mediated NF-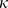
B activation [119]. Similarly, we have got *c*-value of 0 for NF-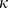
B in an unperturbed signaling pathway, whereas it has become 0.2298 in a perturbed one. It has again become lower (0.19048), while applying conflicting objective function optimization.

Similarly, Wang et al. [117] has shown that CARMA1 functions as a scaffold protein to recruit PKC-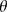, Bcl10, and IKK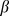 to the lipid rafts of the immunological synapse, which ultimately leads to the activation of NF-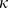
B. A study by Narayan et al. [120] showed that CARMA complex is required for the induction of NF-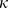
B by AKT, along with PKC activation using a CARMA1-deficient T cell line. AKT has a role in NF-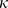
B induction in T cells [121]. It is here to be mentioned for information that AKT acts upstream of the IKK complex to increase IKK activation, I
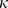
B degradation and NF-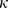
B nuclear entry [122]. The *c*-values for AKT1 was lower (0.032244) in an unperturbed TCR signaling pathway, whereas, it was higher (0.95152) in perturbed one and again lower (0.093664) in an integrated pathway which was optimized for two conflicting objective functions. The case is similar with IKK complex, where *c*-value is lower (0.70419) in an unperturbed TCR signaling pathway, which later has increased (0.90608) in a perturbed TCR signaling pathway. It has again decreased (0.71188) in an integrated pathway which was optimized for two conflicting objective functions.

MALT1 is recruited to the lipid rafts of the immunological synapse following activation of TCR and CD28 coreceptor (CD3/CD28 costimulation). This recruitment is dependent on CARMA1 [116]. MALT1, Bcl10, and CARMA1 form a trimolecular complex. Expression of a MALT1 deletion mutant has shown to completely block the CD3/CD28 costimulation-induced NF-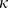
B activation. Considering the above experimental evidences for CARMA complex, we have also found lower (0.39768) *c*-value for CARMA complex in an unperturbed TCR signaling pathway. In contrast, it has become 0.81185 and 0.23229 in pathogen perturbed system and in the system, where it is optimized for two conflicting objective functions, respectively.

NCK plays a pivotal role in TCR-induced reorganization of the actin cytoskeleton and the formation of the immunological synapse in T lymphocytes. It binds with phosphorylated SLP76 and recruits WASP, where, SLP76 functions as a scaffold bringing NCK and WASP into proximity with VAV1 and Cdc42-GTP [123], [124]. TCR-dependent activation of PAK1 and TCR-inducible association of PAK1 with NCK is also evidenced [125]. PAK1 belongs to a family of closely related serine/threonine kinases and its activation contributes to TCR-induced ERK activation, calcium flux and the NFAT transcriptional response [125], [126]. It gets activated upon TCR stimulation and associates with NCK adaptor. Yablonski et al. [125] has shown that the dominant negative alleles of PAK1 or NCK specifically block TCR-induced NFAT activation. From observing quantities of *c*-values obtained from our results, we can say that we have got almost similar results in the form of lower *c*-values of NCK and PAK in an unperturbed TCR signaling pathway, which then has increased upon stimulation. We have found lower (0.654) *c*-value for NCK in an unperturbed TCR signaling pathway. In contrast, it has become 0.57508 and 0.042724 in pathogen perturbed system and in the system, where it is optimized for two conflicting objective functions, respectively. For PAK, the *c*-values are, 0.017261 for an unperturbed TCR signaling pathway. In contrast, it has become 0.20377 and 0.096005 in pathogen perturbed system and in the system, where it is optimized for two conflicting objective functions, respectively.

In activated T cells, SOS promotes the conversion of Ras to the GTP-bound active state [127], whereas VAV is involved in Rac GDP/GTP exchange [128], [129]. Disruption of TCR signaling pathway in VAV-deficient mice had shown decrease in IL-2 secretion and TCR-mediated cytoskeletal reorganization in T cells [130], [131]. VAV-deficient mice had also defects in TCR-induced intracellular calcium fluxes as well as in the activation of MAPK and NF-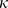
B [132]. One of the study has also shown that phosphorylation of SOS requires expression of CD45 and LCK; and depends upon signaling via PKC [133]. Here, the authors showed that the TCR-induced phosphorylation of SOS does not require activation of MEK/ERK pathway, however, they have found that the basal phosphorylation of SOS in T cells is affected by either MEK or MEK-dependent kinases. In the same way, through *in silico* analysis, we have found lower (0.5286) *c*-value for SOS in unperturbed TCR signaling pathway. In contrast, it became 0.50821 in both pathogen perturbed system and in the system, where it is optimized for two conflicting objective functions, respectively. Considering the case of VAV, we have found lower (0.24826) *c*-value for VAV in an unperturbed TCR signaling pathway. In contrast, it has become 0.7427 and 0.53051 in a pathogen perturbed system and in the system, where it is optimized for two conflicting objective functions, respectively.

## Discussion

We have constructed an integrated pathogen perturbed immune signaling pathway model. Here we have implemented a simple gradient descent based optimization developed under FBA, which enables us to compare and studied the behavior of TCR signaling pathway of *H. sapiens* for aforesaid six different cases. In this way, we can study how an immune signaling pathway behaves at the time of infection/pathogen attack. Significance of this kind of study is that it exposes the proteins which get altered upon infection. The expression of many virulence determinants in *S. aureus* is controlled by regulatory loci, like, agr and sar. *S. aureus* toxins are the staphylococcal enterotoxin, SAgs that can stimulate whole T cell subpopulations by cross-linking TCRs with MHC-II molecules. Both human and mouse T cells can be activated by enterotoxins and produce cytokines.

Superantigen (SAg) stimulates naive T cells *in vivo*. Responder T cells that survived the SAg challenge were found to be incapable of generating a secondary proliferative response (known as anergy) against SAg due to CD8-positive regulatory suppressive T cells. These regulatory cells inhibit cell division of preactivated T cells but not the SAg response of naive T cells [4]. It has been demonstrated in mice that the administration of SEB leads to unresponsiveness in V
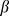
8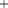 T cells *in vivo* [134]. It then led to the death of up to 50% of V
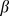
8-positive cells by 7 days after SEB administration. The remaining V
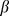
8-positive cells were unresponsive to receptor-mediated cross-linking to TCR [15]. A selective impairment in the TCR-induced activation of the Ca/calcineurin pathway is responsible for the *in vivo* SEB-induced anergic state of T cells [135]. The biochemical state of anergic T cell clones is also characterized by a block in Ras-mediated activation of ERK1/2 and reduced activity of JNK and p38 [136]. In the present article, we optimized and compared some molecules present in TCR signaling pathway of *H. sapiens* under pathogen perturbed and unperturbed conditions, assuming naive T cells.

## Methodology

Systems level modeling and simulations have been proved invaluable in obtaining a quantitative and dynamic perspective of various aspects of cellular functions. Among them, constraint-based analyses, such as flux balance analysis (FBA), are quite popular for simulating cellular systems, specifically metabolism. Unlike mechanistic simulations (that depend on scarcely available accurate kinetic data), FBA is based on the principle of conservation of mass in a network, which utilizes the stoichiometric matrix and a biologically relevant objective function to identify optimal reaction flux distributions.

Stoichiometric representation of a biochemical pathway can be in the form of a matrix, 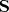, where 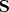 is of the order of 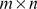. Here, 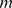 indicates the number of components and 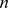 stands for the number of reactions. We have considered a modified formulation for steady state and is given by 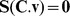 [20], [21]. Biochemical systems are large enough to investigate in terms of participating molecules and interactions present among them. While studying biochemical pathways/networks, under the framework of systems biology, one needs to define the system first by putting an appropriate boundary. Under this scenario, steady state assumption may need to be considered [137], otherwise, concentrations of some of the molecules inside the system may grow indefinitely. It is true that excess concentrations of these molecules leave the system through crosstalk with other pathways outside the system. However, we have not considered here the notion of crosstalk and hence assumed steady state behavior of the reactions inside the system. For example, in the present study we have considered only host TCR signaling pathway that is affected by SAg from *S. aureus*. However, it is possible that some other host’s systems may also be affected due to the pathogen attack. In the case of pathogen *S. aureus*, we have only considered expression regulatory pathway for SAg, although, *S. aureus* expresses many other forms of enterotoxins, exotoxins as well as virulence proteins. Therefore, steady state and closed boundary assumptions are required here.

For the expression 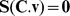, it is to be mentioned here that the concentration of molecules (*e.g.*, transcription factors/enzymes/other regulators, like, addition of phosphate molecule in phosphorylation reactions, called phosphoregulation), catalyzing various reactions in the biochemical network, may not be expressed at the desired level. This is the reason behind incorporating concentration factor 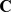 in the form of weighting coefficients. Here, 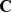 is an 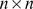 diagonal matrix whose diagonal element are the components of a vector 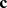. That is, if 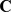 = 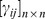, then 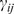 = 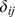 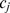, where 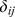 is the Kronecker delta. The term 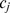 indicates the level of concentration of the molecule(s) catalyzing 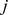
th reaction. In the case of a metabolic pathway, 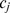 refers to the concentration of the enzyme catalyzing 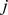
th reaction in the pathway. Here regulators need to be present in the required level of concentration for carrying out reactions/conversions in the integrated host-pathogen interaction pathway. Thus we can draw an analogy between enzyme catalyzing a reaction in a metabolic pathway and regulators associated with a reaction/conversion in the said integrated pathway. However, the number of regulators corresponding to such a reaction/conversion is normally more than one. In order to simplify the scenario, we consider an aggregated concentration of the regulators corresponding to a reaction/conversion in the integrated pathway. Hence, 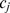 reflects this aggregated concentration of the regulators corresponding to 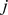
th reaction/conversion in the pathway. Here 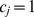 denotes that the required amount of the regulators corresponding to the 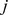
th reaction is available in the cell. In contrast, 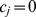 indicates that insufficient amount of the regulators are present for that reaction.

We are able to know, using 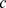-values, the required aggregated concentration levels of the regulators corresponding to certain reactions in the integrated pathway at a given condition in a cell. In the present study, we have optimized (maximized or minimized) the concentrations of some molecules either in the pathogen or in the host. In other words, we want to estimate optimal aggregated concentrations levels (or expression levels), *i.e.*, 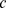-values, of the regulators so that the concentrations of some molecules in the integrated pathways become maximum and/or minimum. We have kept manipulating the expression levels of specific regulators in different cases to investigate their effects on the behavior of host’s immune system at the time of pathogen attack. We have done this in the form of implementing various objective functions for 6 different cases.

We would like to further elaborate on the biological significance of 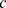-values illustrating an example from the present study. In case (4), we have optimized two conflicting objective functions simultaneously, *viz.*, minimization of the expression level of SAg in *S. aureus* and maximization of the expression/concentration levels of TCR:CD3 complex and ZAP70 in host. Minimization of the expression level of SAg in *S. aureus* simply refers to *in silico* manipulation of the concentration of the regulators (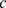-value) that is responsible for its expression. Similarly, it is done for maximization of the expression/concentrations of TCR:CD3 complex and ZAP70 in the host.

In FBA, we take the algebraic sum over the reactions 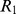, 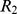,…,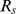 that directly yield the target molecule, and is given by equation, *i.e.*, 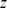 = 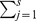 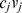. It needs to be maximized and/or minimized according to the problem. For generating flux vectors, we generate the basis vectors 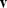 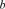 and the number of such basis vectors is 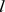 [20], [21]. Then we generate 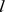 number of positive random numbers 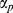, 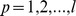, and finally, we generate flux vectors 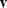 in the form of linear combinations of the basis vectors using 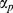, *i.e.*, 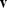 = 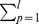 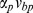. All the internal fluxes are non-negative. The constraints on the exchange fluxes depend on their directions and can be expressed as 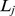 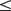 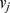 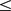 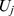, 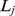 and 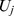 being lower and upper bound of 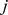
th exchange flux [138]. The fluxes in a metabolic pathway represent the rate of mass flow from one metabolite to the other through a reaction. The flux vectors are replaced by signal flow vectors in the context of a signaling pathway. Thus the objective function becomes
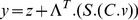
(1)


Here 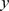 needs to be minimized with respect to the weighting factors 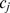 for all 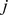. The term 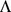 = 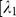, 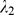, 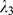,…, 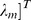 is the regularizing parameter. We consider 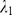 = … = 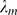 = 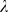 for simplicity. Here 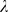 has been chosen empirically as in [20].

We have used gradient descent technique for minimization of 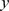 [139]. Initially, a set of random values in [0, 1] corresponding to 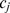
’s are generated. These 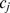
’s are then modified iteratively using the gradient descent technique, where the amount of modification for 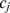 in each iteration is defined as
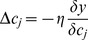
(2)


The term 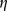 is a small positive quantity indicating the rate of modification. For computing the values of 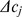
’s, we use the following expression
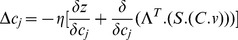
(3)


### Development of a Conflicting Objective Function Optimization Algorithm under FBA

It is very rare to find a solution that simultaneously optimizes all the objective functions in conflicting optimization problems. Here, the problem is to consider conflicting objectives (minimization of an objective function, while maximizing the other one), *e.g.*, to minimize the toxin expression/production in a pathogen and simultaneously maximize the concentration of defense molecules of an immune system signaling of an infected host with respect to 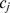
’s. Now, the expression for 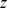 becomes 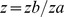, where 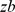 is to be minimized and 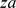 requires to be maximized. Weighting coefficients 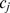 for this purpose are estimated. The expression given in equation (3) is modified into the following expression,
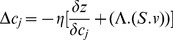
(4)


The modified value of 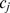 is given by
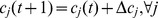
(5)where 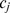 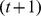 is the value of 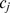 at iteration 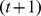, which is computed based on 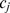-value at iteration 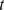. We set the number of iterations and observe how objective functions are optimized with iterations. It has been shown in Fig. 7.
